# Application of 3-D Urbanization Index to Assess Impact of Urbanization on Air Temperature

**DOI:** 10.1038/srep24351

**Published:** 2016-04-15

**Authors:** Chih-Da Wu, Shih-Chun Candice Lung

**Affiliations:** 1 Department of Forestry and Natural Resources, National Chiayi University, Chiayi, Taiwan; 2 Research Center for Environmental Changes, Academia Sinica, Taipei, Taiwan; 3 Department of Atmospheric Sciences, National Taiwan University, Taipei, Taiwan; 4 Institute of Environmental Health, School of Public Health, National Taiwan University, Taipei, Taiwan

## Abstract

The lack of appropriate methodologies and indicators to quantify three-dimensional (3-D) building constructions poses challenges to authorities and urban planners when formulating polices to reduce health risks due to heat stress. This study evaluated the applicability of an innovative three-dimensional Urbanization Index (3DUI), based on remote sensing database, with a 5 m spatial resolution of 3-D man-made constructions to representing intra-urban variability of air temperature by assessing correlation of 3DUI with air temperature from a 3-D perspective. The results showed robust high correlation coefficients, ranging from 0.83 to 0.85, obtained within the 1,000 m circular buffer around weather stations regardless of season, year, or spatial location. Our findings demonstrated not only the strength of 3DUI in representing intra-urban air-temperature variability, but also its great potential for heat stress assessment within cities. In view of the maximum correlation between building volumes within the 1,000 m circular buffer and ambient air temperature, urban planning should consider setting ceilings for man-made construction volume in each 2 × 2 km^2^ residential community for thermal environment regulation, especially in Asian metropolis with high population density in city centers.

Human activities in urban areas affect the Earth’s energy budget by changing land surface properties and pollution emissions. The resulting climate change affects daily weather conditions. The enhanced temperatures have caused significant health impacts, especially during heat waves^1^. For example, the 2003 European heat wave was the hottest summer on record in Europe since at least 1540^2^. Between June and August, 2003, air temperature hit record high in various cities and countries, reaching as high as 48 °C in Amareleja, Portugal. The disastrous heat wave led to health crises, claiming more than 70,000 lives^3^. Extreme weather events also had great impacts on human health in Asia. Wu *et al*. used temperature in Taipei above 35 °C as a cut-off point for defining heat waves and found that mortality increased significantly island-wide during heat waves in 1994–2003 with evident spatial variability^4^.

Urban areas that hold more than half the world’s population were blamed for the majority of greenhouse gas emissions for global warming^5^. Urban Heat Island (UHI) effect resulted from urbanization which involves concentrated energy consumption and dense urban infrastructures also aggravates the temperature increase in urban areas under climate change^6^. To improve the understanding of the association between ambient air temperature and built-up factors, such as built-up ratio, spatial pattern of land-use types, and building heights, is important for establishing environmental health adaptation and hazard prevention strategies under climate change impacts. Up to now, there are few studies considering three-dimensional (3-D) man-made constructions in intra-urban temperature assessment^7^. In our previous work, an innovative three-dimensional Urbanization Index (3DUI) was developed to quantify urbanization, based on remote-sensing database, from a 3-D perspective with a 5 m spatial resolution taking into account the total volume of human constructions^8^. Correlation coefficients of medium to high levels (>0.6) were found between 3DUI and spectrum-based surface temperatures.

Built on previous findings, this study further compares 3DUI with on-site air temperature monitoring records to confirm the representativeness of 3DUI on the spatial variability of air temperature in urban areas, which has direct impacts on human health. The objective of this study is to evaluate the applicability of 3DUI in representing intra-urban variability of air temperature by assessing correlation of 3DUI with air temperature in urban areas. The evaluation involves the following three steps. First, the correlation between 3DUI and air temperature was assessed. Second, the correlation coefficients obtained from various circular buffer zones surrounding the air temperature monitoring stations were compared to determine the buffer zone with the highest correlation. The choice of zone size is particularly important for authorities when formulating environmental health adaptation strategies to minimize heat stress in residential communities. Finally, whether spatial (different areas and cities), temporal (different years and seasons), or environmental (e.g., vegetation and water) factors would affect the correlation estimates were investigated.

## Results

### Relations between 3DUI and air temperature

**Figure 1:**
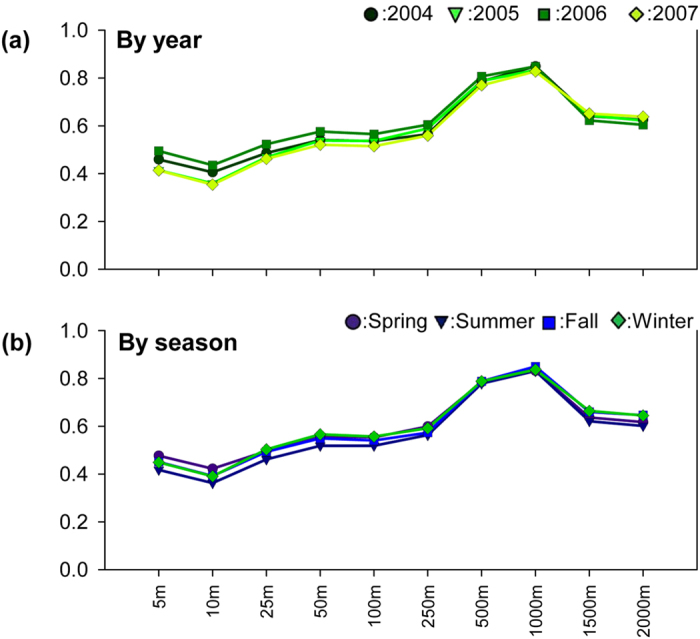
Spearman correlation coefficients between (**a**) annual and (**b**) seasonal average of air temperature and 3DUI with various buffer distances from weather stations located in the Taipei metropolis (*p* < 0.05 in all cases).

Figure 1(a,b) shows respectively the Spearman correlation coefficients between annual and seasonal average of air temperature and 3DUI at various buffer distances from the weather stations located in the Taipei metropolis. The correlation coefficients are all statistically significant (*p* < 0.05), showing overall the same trend regardless of year or season and marked difference in estimates for various buffer sizes. As seen in Fig. 1(a), the correlation estimates for each buffer zone from 2004 to 2007 were either the same or close, showing small variations over the years. For example, the coefficients derived for the 5 m buffer zone ranged from 0.41 to 0.5 in these four years. Moreover, the estimates were increasing with increase in size of buffer from 5 m to 1,000 m, followed by decrease with further enlarging of buffer size. In other words, the correlation coefficients peaked at 1,000 m (0.83 to 0.85) from the weather station, implying that the total volume of man-made constructions within the 1,000 m circular zone had the highest correlation with annual air temperatures measured at the weather stations. The same observation is found for the data when stratified by seasons. As shown in Fig. 1(b), the highest coefficient estimates, ranging from 0.83 to 0.85, are also found in the 1,000 m buffer zone. The consistent findings for both annual and seasonal averages of air temperature demonstrate that 3DUI is a good indicator for assessing the urban thermal environment.

### Sensitivity analysis results

**Figure 2:**
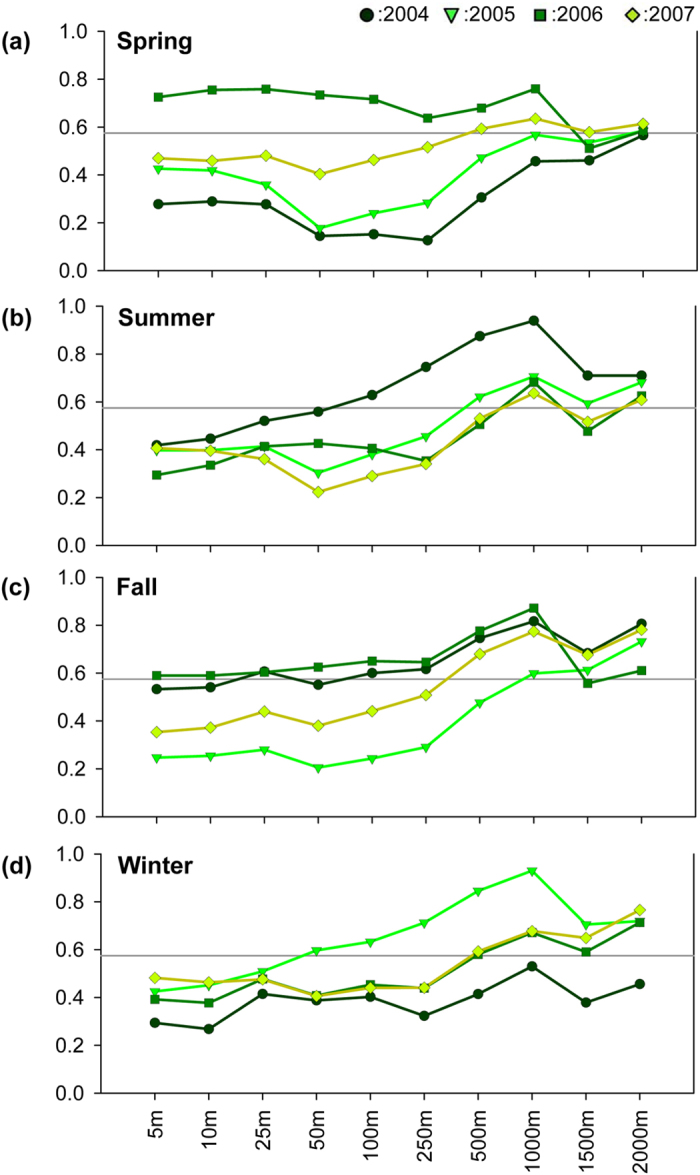
Spearman correlation coefficients of air temperature and 3 DUI for weather stations in Taipei City in (**a**) spring, (**b**) summer, (**c**) fall, and (**d**) winter. (*p* < 0.05 in all cases above the horizontal line).

**Figure 3:**
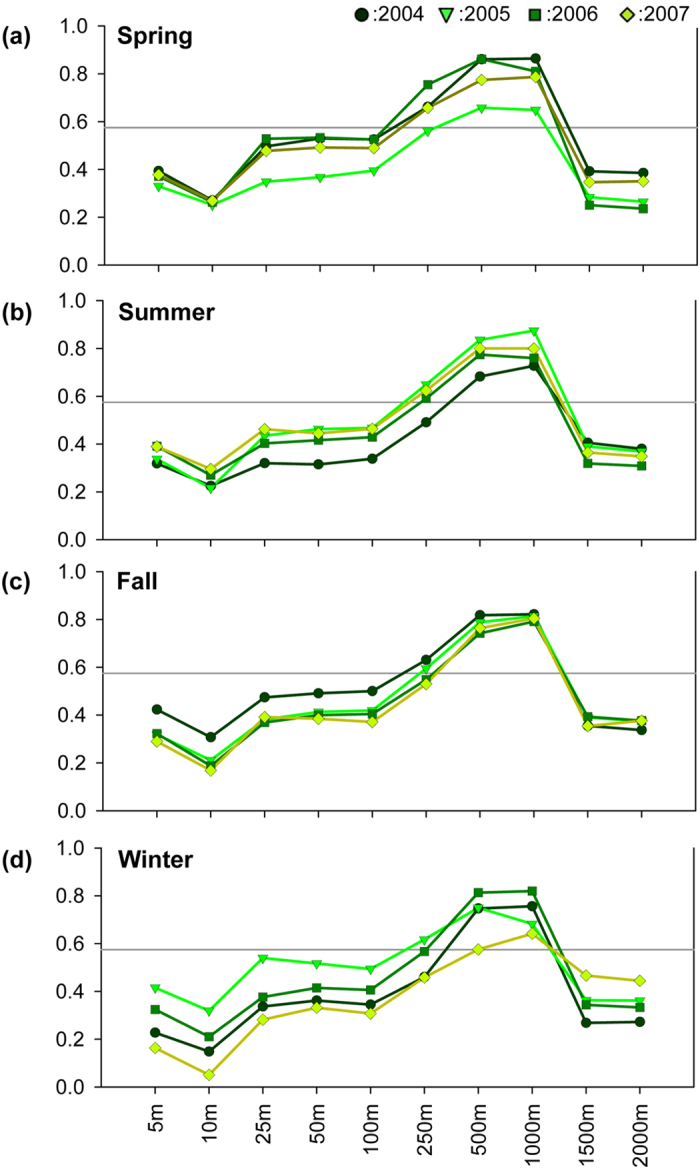
Spearman correlation coefficients of air temperature and 3 DUI for weather stations in New Taipei City in (**a**) spring, (**b**) summer, (**c**) fall, and (**d**) winter. (*p* < 0.05 in all cases above the horizontal line).

Figures 2 and 3 show the correlation coefficients of seasonal averages of air temperature and 3DUI at various buffer distances from the weather stations located in Taipei City and New Taipei City, respectively. Similar patterns as those in Fig. 1 can be seen; that is, a trend of increase in correlation between air temperature and 3DUI which peaks at 1,000 m buffer distance, followed by a slight decline. Besides seasonal variations between the two studied areas, some coefficients also became statistically insignificant due to the reduced sample size.

Table 1 shows the correlation coefficient estimates obtained when stations in proximity of main streams and those surrounded by abundant vegetation are excluded to examine the effects of environmental factors. In general, medium to high correlations (0.61 to 0.88) were observed. The consistently strong correlation indicated the representativeness of 3DUI on thermal environment within 1,000 m from weather stations located in various surroundings with water and vegetation.

### Spatial variability of 3DUI within Taipei metropolis

**Figure 4:**
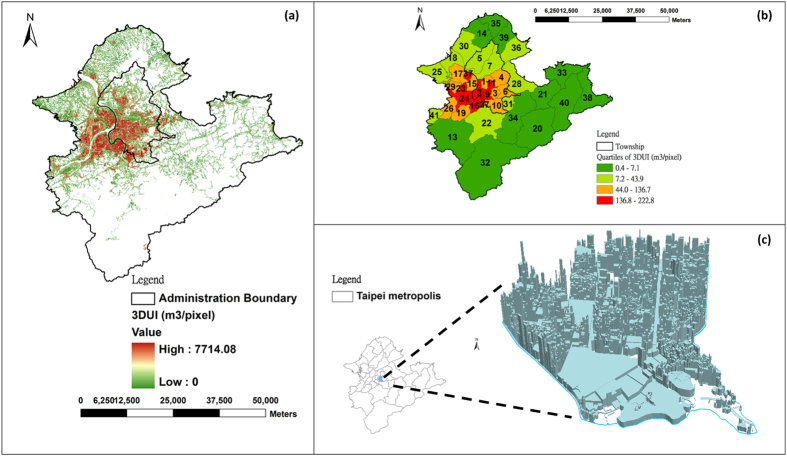
Illustration of 3DUI at various spatial scales. (**a**) city, (**b**) township (mean 3DUI value of each township), and (**c**) community. Esri ArcGIS 10.2 and ArcScene 10.2 were used to create this figure (http://www.esri.com/software/arcgis).

Figure 4 illustrates the variability of 3DUI within the study area at different spatial scales. Shown in Fig. 4(a) are areas of the whole Taipei metropolis marked in red and green denoting respectively high (7,714 m^3^/pixel) and low (1 m^3^/pixel) 3DUI. Areas marked in white have natural land cover with 3DUI assigned as 0. As can be seen, areas with high 3DUI indicating intense human constructions formed a spatial cluster at the center of the Taipei metropolis while there are scattered plots of high 3DUI in its outer ring. Such spatial distribution of 3DUI not only evidences concentration of human constructions within urban and suburban areas, but that pressures from urban sprawl and population explosion are also changing rural spaces of the Taipei metropolis with individual settlements sprouting in relatively natural areas. The estimated mean 3DUI values for the whole Taipei metropolis, Taipei City, and New Taipei City were 24.7, 76.9 and 17.8 m^3^/pixel, respectively; with that of Taipei City almost four times that of New Taipei City. This huge discrepancy in 3DUI would be reflected in the two areas experiencing different intensity of UHI effects.

Table 2 lists the mean 3DUI value of each township in the study area and Fig. 4(b) shows the mean 3DUI values at township resolution. As can be seen, mean 3DUI values of townships spread over a wide range from 0.4 m^3^/pixel (townships covered by natural land forms with minimum 3DUI) to 222.8 m^3^/pixel (townships having dense high-rise constructions with maximum 3DUI), demonstrating huge difference among townships. Moreover, townships with mean 3DUI values higher than the third quartile are again concentrated in the heart of the Taipei metropolis surrounded by townships with mean 3DUI values lower than the second quartile, mostly located in its periphery. Fig. 4(c) is the 3-D illustration of 3DUI at the community scale, showing 3-D urban structures at 5 m resolution. Taken together, the illustrations at different spatial scales displayed in Fig. 4 clearly reveal that 3DUI is a good indicator for quantifying 3-D urban structures not only at the city scale, but also for examining intra-urban variations. Moreover, the numerical values of 3DUI can also serve as an indicator for assessing the impacts of urbanization, such as UHI effects.

## Discussion

Extreme temperatures have significant impacts on human health. For example, Chung *et al*.^9^ examined the impact of heat waves on mortality in Taipei, Taiwan from 1994 to 2003. They found that respiratory mortality increased by 9.3% (confidence interval (C.I.) 4.1–14.8) per 1 °C increase when air temperature rose above 31.5 °C; while cardiovascular mortality increased by 1.1% (C.I. 0.3–1.9) per 1 °C increase when ground air temperature was above 25.2 °C. In recent years, the air temperature in Taipei has frequently exceeded 35 °C, implying significant increases in respiratory and cardiovascular mortalities on hot days. In urban areas, UHI effects due to urbanization push up temperatures. Wu *et al*.^8^ had obtained positive correlation coefficients between 3DUI and surface temperature up to 0.83. This study further demonstrated strong correlation of 3DUI with air temperature, which is directly related to heat stress experienced by human beings^9^. The current results again show that 3DUI can be adopted as a good indicator for outdoor thermal environment assessment with fine resolution, and be in turn applied to heat-related health risk assessment in urban areas.

Compared with Taipei metropolis as a whole (Fig. 1), Taipei City and New Taipei City, when assessed separately (Figs 2 and 3), showed variations in correlation coefficients among seasons and years, with Taipei City having larger variations than New Taipei City. Such difference can be attributed to sample size; there were fewer observation stations in Taipei City (n = 12) than in New Taipei City (n = 20). Some of the correlation coefficients became insignificant also due to smaller sample size. Nevertheless, the 1,000 m buffer zone had the highest correlation in most cases. In other words, despite the variations, the choice of best buffer size is not affected by the spatial scale, as in Taipei metropolis versus Taipei City and New Taipei City. However, the coefficients themselves are very sensitive to sample size. Hence, to obtain robust estimation results, there should be enough samples and the sample size cannot be too small.

Several remotely sensed indices including Normalized Difference Vegetation Index (NDVI) and Normalized Difference Built-up Index (NDBI) were employed in previous studies to assess urbanization from 2-D perspective. For example, Wang *et al*.^10^, examined temporal relationships of remotely sensed Advanced Very High Resolution Radiometer (AVHRR) NDVI with temperature in Kansas during a nine-year period (1989 to 1997). The results showed that air temperature was positively correlated with NDVI both early and late in the growing season (correlation coefficients ranging from 0.11 to 0.87), and there was a weak negative correlation between temperature and NDVI in the middle of the growing season (correlation coefficients ranging from −0.69 to −0.02). Moreover, instead of air temperature, most studies focused on the relations between NDBI and Land Surface Temperature (LST). Liu and Zhang^11^ used the Landsat TM and ASTER data of Hong Kong in 2005 to assess the correlations of LST with NDVI and NDBI. They found that the correlation coefficient of LST with NDVI is −0.41, while the coefficient of LST with NDBI is 0.71. Compared with the above two approaches, the proposed 3DUI using a 3-D methodology showed high correlation with air temperature in all four seasons (correlation coefficients ranging from 0.83 to 0.85 in the 1000 m buffer zone). In addition, the spatial resolution of the satellite images used for calculating the 2-D urbanization indices, such as NDVI and NDBI in the above two approaches, was 1.1 km for NOAA AVHRR, 30 m for Landsat TM, and 15 m for ASTER. In this study, 3DUI provides a quantitative measurement of 3-D urban structures with a 5 m spatial resolution. Thus, 3DUI has a finer spatial resolution but maintains the advantages of studying a vast area. The high correlation with air temperatures shown in this study was mainly due to the 5 m fine resolution of 3DUI with the capacity to depict 3-D urban structures. Since ambient air temperature is related to outdoor heat stress experienced by residents in communities, 3DUI could be applied for heat stress assessment. Moreover, the representativeness of 3DUI for UHI effects will not be affected by environmental factors such as vegetation and water. These features demonstrate the advantages and applicability of 3DUI for UHI assessment under various environmental conditions.

In this study, 10 different buffer sizes ranging from 5 m to 2,000 m were employed to examine the spatial effects on relations between 3DUI and air temperature. This sensitivity test has never been done in previous studies. The results showed that building volumes within 1,000 m circular buffer distance had the strongest correlation with ambient air temperature, indicating that 3-D urban structures within 1,000 m circular area have strong direct impacts on ambient air temperature of the same area. Identifying this zone size (1,000 m circular area) is particularly important for authorities when formulating environmental health adaptation strategies to minimize heat stress. In actual practice, a circular area of 1,000 m can be viewed as a 2 × 2 km^2^ urban planning cell. In view of the present findings, government authorities and city planners should consider setting a ceiling for man-made constructions in a spatial scale of 2 × 2 km^2^ for residential communities. Such measure is particularly useful for populous metropolises. Asian cities usually have high population density; with residential communities surrounded by high-rise commercial blocks and most urban residents dwelling in multi-story apartment buildings. Rapid economic development leads to ever-increasing demand for land and man-made constructions continue to replace green spaces in core urban areas, thus affecting the thermal environment in urban ecology. Increase in impervious surface intensifies UHI effects, further aggravating urban warming. Urban vulnerable populations like the elderly, children or patients are at risk and subject to the health impacts from UHI effect on top of climate warming. Hence, there should be regulations on the maximum volume of man-made constructions allowed in places where these vulnerable populations live and gather, such as kindergartens, elementary schools, hospitals, and nursing homes.

To moderate ambient air temperature and UHI effects, a ceiling for volume of man-made constructions should be set in each 2 × 2 km^2^ residential community and green spaces should be preserved instead of being turned into impervious surfaces. Urban green spaces and facilities that can encourage physical activities and social contacts should be provided to reduce psychophysiological stress and depression, not to mention that measures should be taken to decrease noise and air pollution^11^,^12^,^13^. The present findings provided crucial scientific support on the importance of planning on a small community scale (2 × 2 km^2^) rather than at the entire city level, especially when formulating environmental health adaptation strategies. After all, heat stress residents experienced comes more from community thermal conditions than city-wide temperature averages, in particular for cities with large intra-urban temperature variability.

## Methods

### Study area and databases

**Figure 5:**
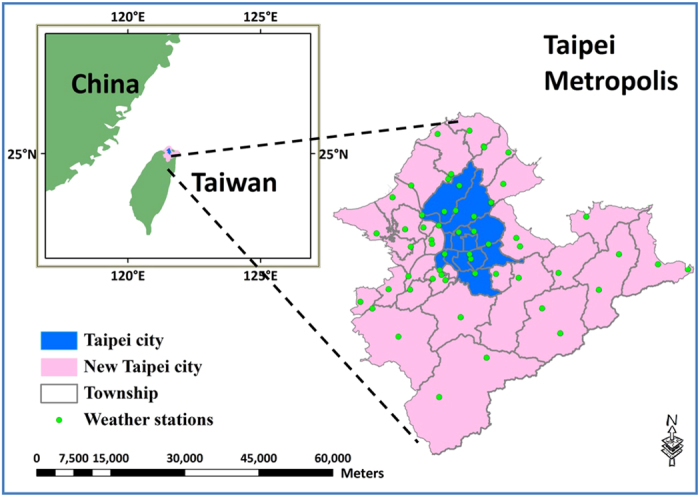
Location of Taipei metropolis and weather stations. The source data of this figure is the township map of Taiwan obtained from the Ministry of Interior, Taiwan. Esri ArcGIS 10.2 was used to create this figure (http://www.esri.com/software/arcgis).

The selected study area is the Taipei metropolis (Fig. 5). Taipei metropolis, which consists of Taipei City (the capital of Taiwan) and New Taipei City, was selected as the study area. It comprises 41 townships and 1,488 census tracts, stretching over a total area of 2,326.5 km^2^. The population density of Taipei City and New Taipei City are 9919 people/km^2^ and 1929 people/km^2^, respectively. The average number of residents per census tract is 5,710 for Taipei City and 3,579 for New Taipei City^14^. In general, more than 25% of Taiwan’s population lives in Taipei metropolis (approximately 6.6 million with an average density of 2,863 people/km^2^) because of more job opportunities in this area. As for land-use types, 13.2% of the Taipei metropolis is classified as of impermeable surface (e.g., buildings and roads) and 68.33% is covered by forest^15^.

Four spatial databases were used in this study, namely Digital Terrain Models (DTMs), national land-use inventory, global MODIS Normalized Difference Vegetation Index (NDVI) database, and geographic information system (GIS) layer of river network. DTM is a mathematical representation of topography^16^, and can be classified into two categories, Digital Elevation Model (DEM) and Digital Surface Model (DSM). DEM consists of terrain elevations for natural ground surfaces at regularly spaced horizontal intervals^17^. While DSM not only incorporates natural ground surfaces, it also takes into account buildings and other objects higher than the underlying topographic surfaces, such as trees and rooftops^18^. Processed from aerial photos taken in 2004–2005, both DEM and DSM of 5 m resolution were employed to establish the 3DUI^8^; their horizontal and vertical variations in urban area are less than 0.5 m and 0.7 m, respectively^19^. The national land-use inventory generated using multisource images (including aerial photos and satellite images) collected from 2006 to 2007 was acquired^15^. Land-use types including constructions for transportation, recreation, public and private buildings were selected as human constructions for establishing the 3DUI. The amounts of trees and vegetation (green spaces) in the studied areas were obtained from NASA’s Earth Observing System data – the global Moderate Resolution Imaging Spectroradiometer (MODIS) NDVI. This system calculates the global distribution of vegetation types, as well as their biophysical and structural properties and spatial/temporal variations. NASA provides Global NDVI data updates every 16 days at 250 m spatial resolution as a gridded product in the sinusoidal projection^12^,^20^. A total of 48 NDVI images (one per month) were derived from the U.S. Geological Survey (USGS) from 2004 to 2007. The spatial mean of NDVI of each township was employed to quantify the greenness surrounding each weather station. Stations with NDVI values exceeding the third quartile were determined as “greenest” weather stations. The GIS map of river network of Taiwan obtained from the Academia Sinica Computing Center was adopted to assess the proximity of each weather station to the main streams. The NDVI and river network databases were employed to examine the effects of vegetation and water, respectively, on the robustness of the statistical estimates. Finally, ArcGIS 10.2 and SAS 9.3 were used for data analysis. Moreover, daily air temperature records from 2004–2007 were acquired from 32 meteorological stations of the Central Weather Bureau located in Taipei metropolis. Mean annual temperature of Taipei metropolis was calculated according to these daily observations. Furthermore, March to May, June to August, September to November, and December to February were defined as spring, summer, fall, and winter, respectively. Mean seasonal temperature was then calculated for the whole Taipei metropolis, as well as Taipei City and New Taipei City respectively.

### Calculation of 3DUI

3DUI is a quantitative 3-D urbanization index, which takes into account the total volume of human constructions of an area, including building and transportation constructions. The accuracy of 3DUI has been validated and proven in earlier research^8^. Briefly, the heights of human constructions were calculated by subtracting the elevation values in DEM from those in DSM for the man-made land-use types in the national land-use inventory. A constant of 0.2 m was adopted as the thickness of roads according to the “Regulations of Urban Road Designs”^19^ since man-made road surfaces can absorb heat and contribute to UHI. Finally, the results were multiplied by the size of pixel (5 m × 5 m = 25 m^2^) to derive the total volume of human constructions for 3-D urbanization assessment. In other words, the physical meaning of 3DUI value is the volume of man-made construction on a 25 m^2^ pixel; with a large 3DUI indicating intense man-made constructions in the area. Values of pixels with natural land covers (i.e., vegetation, forest, and water) are assigned as “0” because there was no human construction in these areas. In this study, DEM and DSM of 5 m resolution were employed to estimate the 3DUI for the whole Taipei metropolis. Spatial variability of 3DUI was then examined between cities (Taipei City and New Taipei City) and among townships.

### Statistical analysis

Circular buffers at a distance of 5, 10, 25, 50, 100, 250, 500, 1,000, 1,500, and 2,000 m around the weather stations were generated. There are a total of 32 weather stations in the Taipei metropolis, including 12 stations in Taipei city and 20 stations in New Taipei city. Spatial average of 3DUI within each buffer zone was calculated. The data were then stratified by years and seasons, and the Spearman’s rank correlation was employed to assess the association between 3DUI and air temperature. In-depth analysis was made on the buffer zone with the highest correlation between human constructions and air temperature.

### Sensitivity analysis

The effects of spatial and environmental factors on the correlation estimates were assessed. For spatial factors, correlation coefficients obtained from different cities (Taipei City and New Taipei City) and different spatial scales (the whole Taipei metropolis versus Taipei City and New Taipei City individually) were compared. Recent studies have indicated that water and vegetation can regulate the micro-climate and affect the thermal environment^21^,^22^,^23^; hence, weather stations in proximity to the three main streams (within 1000 m buffer zone of Danshui river, Keelung river, and Hsintien creek), and weather stations surrounded by abundant vegetation (NDVI values exceeding the third quartile) were excluded when calculating the correlation coefficients. The results were then compared to confirm the robustness of correlation estimates.

## Additional Information

**How to cite this article**: Wu, C.-D. and Lung, S.-C. C. Application of 3-D Urbanization Index to Assess Impact of Urbanization on Air Temperature. *Sci. Rep*. **6**, 24351; doi: 10.1038/srep24351 (2016).

## Figures and Tables

**Table 1:** Effects of environmental factors on correlation coefficient estimates.

Year/Season		All stations (32)^a^	Stations in proximity of main streams excluded (29)	Stations surrounded by abundant vegetation excluded (24)
2004	Spring	0.83^b^	0.83	0.76
	Summer	0.82	0.79	0.80
	Fall	0.86	0.83	0.87
	Winter	0.75	0.74	0.77
2005	Spring	0.75	0.74	0.83
	Summer	0.85	0.83	0.83
	Fall	0.82	0.81	0.82
	Winter	0.72	0.74	0.61
2006	Spring	0.80	0.79	0.76
	Summer	0.82	0.80	0.79
	Fall	0.84	0.81	0.88
	Winter	0.83	0.82	0.82
2007	Spring	0.83	0.81	0.82
	Summer	0.77	0.75	0.72
	Fall	0.79	0.77	0.78
	Winter	0.79	0.79	0.73

Number in the parenthesis denotes the number of weather stations included in the analysis. *p* < 0.05 in all cases.

**Table 2:** Mean 3DUI value of each township in Taipei City and New Taipei City.

City	Town	Area (km^2^)	Mean 3DUI (m^3^/25m^2^)
Taipei City	1	13.8	134.5
	2	7.5	198.0
	3	11.2	136.7
	4	32.0	59.9
	5	57.7	34.5
	6	21.9	57.4
	7	62.1	43.9
	8	4.8	173.3
	9	11.3	178.8
	10	31.5	91.3
	11	8.9	138.9
	12	7.0	146.3
New Taipei City	13	189.5	5.7
	14	66.4	6.5
	15	18.1	129.2
	16	19.2	156.8
	17	34.3	51.2
	18	38.7	21.5
	19	29.9	73.1
	20	168.3	0.5
	21	71.2	1.6
	22	121.2	27.7
	23	20.7	157.0
	24	21.5	172.0
	25	53.7	21.4
	26	32.0	59.9
	27	6.2	222.8
	28	71.7	32.0
	29	18.1	65.6
	30	72.1	27.1
	31	21.0	19.7
	32	333.4	0.4
	33	71.5	7.1
	34	141.8	1.2
	35	52.3	3.0
	36	62.9	9.5
	37	7.7	156.2
	38	98.1	2.8
	39	47.1	3.4
	40	146.1	0.9
	41	21.9	42.0

The townships are numbered accordingly to the labels in Fig. 4(b).
